# Animal Toxins as Therapeutic Tools to Treat Neurodegenerative Diseases

**DOI:** 10.3389/fphar.2018.00145

**Published:** 2018-02-23

**Authors:** Jessica M. de Souza, Bruno D. C. Goncalves, Marcus V. Gomez, Luciene B. Vieira, Fabiola M. Ribeiro

**Affiliations:** ^1^Department of Biochemistry and Immunology, Institute of Biological Sciences, Universidade Federal de Minas Gerais, Belo Horizonte, Brazil; ^2^Department of Pharmacology, Institute of Biological Sciences, Universidade Federal de Minas Gerais, Belo Horizonte, Brazil; ^3^Department of Neurotransmitters, Instituto de Ensino e Pesquisa Santa Casa, Belo Horizonte, Brazil

**Keywords:** neurodegenerative disease, neuronal death, neuroinflammation, excitotoxicity, animal venom, toxins

## Abstract

Neurodegenerative diseases affect millions of individuals worldwide. So far, no disease-modifying drug is available to treat patients, making the search for effective drugs an urgent need. Neurodegeneration is triggered by the activation of several cellular processes, including oxidative stress, mitochondrial impairment, neuroinflammation, aging, aggregate formation, glutamatergic excitotoxicity, and apoptosis. Therefore, many research groups aim to identify drugs that may inhibit one or more of these events leading to neuronal cell death. Venoms are fruitful natural sources of new molecules, which have been relentlessly enhanced by evolution through natural selection. Several studies indicate that venom components can exhibit selectivity and affinity for a wide variety of targets in mammalian systems. For instance, an expressive number of natural peptides identified in venoms from animals, such as snakes, scorpions, bees, and spiders, were shown to lessen inflammation, regulate glutamate release, modify neurotransmitter levels, block ion channel activation, decrease the number of protein aggregates, and increase the levels of neuroprotective factors. Thus, these venom components hold potential as therapeutic tools to slow or even halt neurodegeneration. However, there are many technological issues to overcome, as venom peptides are hard to obtain and characterize and the amount obtained from natural sources is insufficient to perform all the necessary experiments and tests. Fortunately, technological improvements regarding heterologous protein expression, as well as peptide chemical synthesis will help to provide enough quantities and allow chemical and pharmacological enhancements of these natural occurring compounds. Thus, the main focus of this review is to highlight the most promising studies evaluating animal toxins as therapeutic tools to treat a wide variety of neurodegenerative conditions, including Alzheimer’s disease, Parkinson’s disease, brain ischemia, glaucoma, amyotrophic lateral sclerosis, and multiple sclerosis.

## Animal Toxins

Living organisms have to adapt to the different environments and face competition from other creatures, as all species are challenged by natural selection (Darwin, 1859). In the course of evolution, species equipped with specific traits that conferred some kind of benefit regarding their basic needs have advantage over others. Venoms and toxins are therefore products of natural selection. Accordingly, toxins and venoms have evolved independently and can be found in several different taxa, including plants, microbes, cnidarians, mollusks, arthropods, and reptiles. These venoms contain different molecules, including inorganic ions, proteins, nucleotides and enzymes, and can trigger a wide variety of effects, such as hemorrhage, necrosis or neurotoxicity (Fry et al., 2009). Organisms, in most cases, evolved their venoms as part of predatory and defensive strategies, on a continuous long term coevolutionary process. For instance, resistance to snake venom by some of the prey or predators of snakes arouse several times in the course of evolution (Biardi and Coss, 2011; Jansa and Voss, 2011). As analysis of different species reveals a great molecular diversity regarding physiological elements such as enzymes, channels, receptors and other potential targets for toxins, it is easy to understand that evolution can be held responsible for such diversified composition of venoms targeting these elements. Toxins present in these venoms are very specific and potent (Zhang, 2015). Moreover, their components effects are often synergic, an important adaptation to reduce the amount of venom dispensed, considering the elevated metabolic cost of venom production (Nisani et al., 2007, 2012).

Humans have evolved together with venomous animals, and as such, many animal toxins have coevolved with our species. Moreover, animal envenomation has been in the past and still is a worldwide issue and a big cause of mortality and morbidity (Kasturiratne et al., 2008; Balhara and Stolbach, 2014). In that way, research on animal venoms and toxins was initially focused on neutralizing venoms effects and treating animal envenomation. However, in 1781, the Italian naturalist Felice Fontana started investigations on the effects of snake venom against blood coagulation. Later, by the 1890s, the first antivenom therapy was developed by the scientists Albert Calmette, Césaire Auguste Phisalix, and Gabriel Bertrand (Calmette, 1894; Phisalix and Bertrand, 1894). However, as the knowledge about the physiology of venoms and toxins expanded, from foe to friend, these substances became valuable resources for scientific research. For many years now natural toxins have been used as research tools. Experiments using a pufferfish toxin were determinant to establish the contribution of Na^+^ and K^+^ channels to the action potential (Narahashi et al., 1969). The isolation of toxins from snake venom, such as α-bungarotoxin and cobratoxin, allowed the successful purification of nicotinic receptors and its subsequent cloning (Changeux et al., 1970). The ω-conotoxin extracted from *Conus* snails, a fish-eating marine snail, was also very important for N-type Ca^2+^ channel research and subgroups of other toxins generated by biotechnological engineering permitted studies of several subtypes of voltage-dependent Ca^2+^ channels (VDCCs) (Olivera et al., 1987; McEnery et al., 1991).

Notably, venoms from several species are under investigation for the treatment of a variety of pathologies, including cardiovascular disorders, pain, cancer, and several neurodegenerative diseases (Brunner et al., 1980; Bowersox et al., 1996; da Silva et al., 2002; Castro et al., 2005; Ye et al., 2016b). The great diversity of the components contained in the venom of several animal species, as well as their high specificity to cell targets, contribute to making the pharmacological research of toxins an interesting field. Therefore, advances in the understanding of neurodegenerative disorders and the consequent emergence of novel drug targets increases the potential for animal toxins as lead candidates for drug development. This review will cover the current available literature regarding the use of natural toxins to develop therapeutic tools to treat neurodegenerative diseases.

## Neurodegeneration

Neurodegeneration is the underlying cause of a wide variety of neurological pathologies, including Alzheimer’s disease (AD), Parkinson’s disease (PD), Huntington’s disease (HD) and cerebral ischemia (Macdonald et al., 1993; Lang and Lozano, 1998; Goedert and Spillantini, 2006; Kalogeris et al., 2012). The pathological mechanisms underlying these diseases are characterized by loss of tissue structure and cell function in selected vulnerable neural systems, which may lead to gradual cognitive and motor deficits, as well as psychiatric disturbance (Yan et al., 2013). Despite tremendous scientific efforts, the complexity of cell death processes and the difficulty to determine disease etiology pose many obstacles to the full understanding of these diseases and to develop disease-modifying therapies.

Neurons may undergo cell death through a variety of mechanisms, including apoptosis, necrosis, and autophagic cell death (Kroemer et al., 2009). The concept of apoptosis was established in Kerr et al. (1972) and is characterized by preservation of cellular ATP levels and the induction of a metabolic pathway leading to cell shrinkage, development of apoptotic bodies and phagocytosis of these cell fragments (Kerr et al., 1972; Richter et al., 1996; Elmore, 2007). Notably, caspases are the central components of the apoptotic response (Thornberry and Lazebnik, 1998; Shi, 2002; Kroemer et al., 2009). Conversely, necrosis is an acute form of cell death. It is characterized by a severe decrease of ATP levels, dispersion of ion gradients, cell dilatation and, ultimately, cell lysis (Kanno et al., 2006; Kroemer et al., 2009). The capture of the cytoplasmic material for mass degradation within autophagosomes occurs in a process called autophagic cell death (Levine and Kroemer, 2008; Booth et al., 2014). However, despite being conceptually different, these cell death mechanisms coexist and share some common features. Moreover, the neuropathological outcome for the CNS insults in animal models of excitotoxicity, cerebral ischemia, target deprivation/axotomy, and in human neurological disorders is not likely to result from a single process or causal mechanism. Numerous cell death stimuli can activate more than one mechanism of cell death depending on the situation, such as the elemental ‘well-being’ of the cell and the severity and duration of the stress (Yan et al., 2013).

Among the pathological processes that trigger cell death, excitotoxicity is particularly relevant, playing a crucial role in several neurodegenerative diseases. Despite intense research into the mechanisms underlying excitotoxicity, the intracellular mechanisms responsible for this type of neuronal cell death are yet to be fully elucidated. Excessive neuronal activation by excitatory neurotransmitters, such as glutamate, is considered to be the primary cause of excitotoxic injury (Garthwaite and Garthwaite, 1986; Marcaida et al., 1995; Nicholls et al., 1999; Arundine and Tymianski, 2004; Quillinan et al., 2016). Glutamate is the major excitatory neurotransmitter in the CNS (Arundine and Tymianski, 2004; Guerriero et al., 2015). There are two types of glutamate receptors: ionotropic, including *N*-methyl-D-aspartate (NMDA), α-amino-3-hydroxy-5-methyl-4-isoxazolepropionic acid (AMPA), and kainate; and mGluRs (Nakanishi and Masu, 1994). Acute CNS insults, such as ischemia and traumatic brain injury, induce intense release of glutamate, triggering overstimulation of glutamate receptors, especially NMDA receptors, and leading to massive influx of ions, in particular Ca^2+^ (Raichle, 1983; Choi, 1994; Quillinan et al., 2016). Destruction of ionic equilibrium depolarizes plasma membrane potential and reduces intracellular pH (Pepe, 2000; Azarias et al., 2011; Surin et al., 2014). Ca^2+^ captured in the mitochondria leads to mitochondrial membrane potential depolarization and disturbance of mitochondrial function (Miyamoto et al., 2005; Wang and Qin, 2010). The excessive activation of ATP-dependent ion pumps leads to ATP depletion and energetic stress, in an effort to reinstate ionic homeostasis (Garland and Halestrap, 1997; Mukherjee et al., 2008). The mechanisms involved in the metabolic response of neurons to excitotoxicity are complex and play a fundamental role in the capacity of the neuron to adapt and reclaim from such an insult.

Neurons are significantly more susceptible to metabolic stress triggered by excitotoxic events. These events can lead to an increase in ROS (Vergun et al., 2003). Although ROS are important intracellular signaling molecules, at high concentrations they can be cytotoxic, leading to oxidative stress (Touyz et al., 2003; Griffiths, 2005). ROS are described as a set of highly reactive molecules derived from oxygen, which contain unpaired valence electrons (Patten et al., 2010; Bolisetty and Jaimes, 2013). Included in the concept of ROS are the free radicals (superoxide, ^●^O_2_^-^, and hydroxyl radical, ^●^OH) and non-radicals (hydrogen peroxide, H_2_O_2_), which originate by both exogenous and endogenous sources (Wu and Yotnda, 2011; Sinha et al., 2013). The major sources of ROS production is the mitochondrial respiratory chain and, in healthy condition, the production of ROS is equitable by different antioxidant processes (Gandhi and Abramov, 2012; Dasuri et al., 2013). Oxidative stress results from a divergence between ROS production and antioxidant defenses, bringing about overaccumulation of ROS. Oxidative stress can, therefore, promote structural damage to DNA, cell membrane injury and alterations in protein structure and function due to protein oxidation (Gandhi and Abramov, 2012). It has been demonstrated that increased levels of ROS play an important role in the pathogenesis of neurodegenerative diseases (Ray et al., 2012; Dasuri et al., 2013). For instance, increased indices of ROS have been found in the postmortem brain tissues from individuals with neurodegenerative disorders, including PD (Giasson et al., 2000a; Dias et al., 2013; Yan et al., 2013), AD (Butterfield et al., 2002; Yan et al., 2013; Hroudova et al., 2014; Zuo et al., 2015) and ALS (Beal et al., 1997; Pedersen et al., 1998). Moreover, ROS-dependent enhanced oxidative alterations of proteins such as α-synuclein in PD, β-amyloid (Aβ) and tau in AD and SOD 1 in ALS may also potentiate protein misfolding and reduce degradation. Thus, the ROS-dependent changes in protein metabolism increase insoluble aggregates or accumulation of protofibrils under pathological conditions, ultimately contributing to neurodegeneration (Giasson et al., 2000b; Horiguchi et al., 2003). Even in the absence of oxidative stress, some proteins possess the potential to cause toxicity leading to cell death. Proteins have to reach a singular tridimensional structure by a complex folding pathway aiming to be functionally active, which is determined by the primary amino acid sequence and the subcellular environment (Anfinsen, 1973). Proteins that are not able to attain the native state are identified as misfolded and targeted by two degradation pathways: molecular chaperones or the UPS (Herczenik and Gebbink, 2008). Damage in the UPS may be caused by the misfolded protein accumulation in the endoplasmic reticulum or failure of the enzymes that belong to the ubiquitin conjugation and deconjugation pathway. As a consequence, damaged USP leads to the accumulation of protein aggregates inside the cell (Berke and Paulson, 2003). Besides, misfolded protein can also be secreted into the extracellular space, leading to the development of extracellular plaques (Friedrich et al., 2010). Formation of oligomers and aggregates occur when a critical concentration of misfolded protein is attained. Depending on the neurodegenerative disease in question, there may be accumulation of aggregated proteins, both inside and outside the cell. This aggregation can generate deleterious cell effects. All the major neurodegenerative diseases including AD, PD and HD are also known as protein misfolding disorders and they show similarities regarding protein aggregation. For instance, high levels of amyloid β can be observed in the brain of individuals with AD (Glenner and Wong, 1984), α-synuclein in PD (Polymeropoulos et al., 1997), and huntingtin in the case of HD (Davies et al., 1997; DiFiglia et al., 1997). However, although it is clear that the type of protein that forms the aggregates are different in each of these neurodegenerative diseases, the most common consequent abnormality is synaptic dysfunction, characterized by dendritic spine loss and reduced post synaptic density, leading to disturbance of network connections and cell death (Selkoe, 2002; Picconi et al., 2012; Sepers and Raymond, 2014).

Using model organisms, such as *Caenorhabditis elegans*, it has been suggested that, as we age, our body progressively loses some mechanisms related to the prevention of accumulation of erroneously folded proteins (Kirstein-Miles and Morimoto, 2010). Corroborating this hypothesis, in the autopsied brain of elderly, the presence of amyloid plaques, NFTs, Lewy bodies, synaptic dystrophy, neuronal loss and decreased brain volume are consistent findings, even though these individuals were not diagnosed with any neurological disease (Elobeid et al., 2016). Therefore, the boundary between pathological neurodegeneration findings and normal aging alterations is not yet clear. Actually, it is unequivocally accepted that aging causes neurodegeneration. Aging is associated with various processes, including loss of protein homeostasis that leads to the development of aggregates and inclusion bodies, DNA damage, lysosomal dysfunction, epigenetic changes and immune dysregulation. These processes, which are consequences of the interaction between genetic predisposition of an individual and his/her exposure to the environment, determine the incidence and prevalence of neurodegeneration, probably in a cell-specific manner. As a consequence, several diseases might develop in accordance with the spatiotemporal distribution of the lesions (Herskind et al., 1996; Brunk and Terman, 2002; Hernandez et al., 2011). The characterization of all the genetic interactions might result in new therapeutic possibilities aiming to modulate both the aging process and age-associated diseases (Johnson et al., 2015; Lardenoije et al., 2015). However, the cause of most neurodegenerative diseases cannot be clearly demonstrated and appear to be sporadic in most cases, posing difficulties for the development of genetic therapies.

Despite all the knowledge acquired so far, regardless of the type or cause of cell death, there are no disease-modifying drugs to treat neurodegenerative diseases. Neuronal cell death occurs as a result of a series of deleterious events not yet fully understood. Knowledge of more mechanisms that underlie neurodegeneration may help the development of alternatives to abort the damage.

## Animal Toxins to Treat Neurodegenerative Diseases

### Alzheimer’s Disease

First described more than 100 years ago by the German scientist Alois Alzheimer, AD is the most prevalent neurodegenerative disease and the leading cause of dementia (Alzheimer, 1907). More than 24 million people in the world exhibit some form of dementia and this number is predicted to double in 20 years (Ferri et al., 2005). AD is the cause of 60–80% of dementia cases and mostly affects people over 65 years of age, leading to death in about 7–10 years of symptoms onset (Plassman et al., 2007). AD is characterized by severe brain atrophy and progressive neuronal death, affecting cognitive brain areas and leading to severe memory impairments, behavioral changes, language and speaking impairment, attention deficits and overall cognitive decline (Johnson et al., 2008; Holtzman et al., 2011). AD has many etiological factors and, in most cases, reliable diagnostics can only be obtained post-mortem (Castellani et al., 2010). A meta-analysis study showed that 9% of individuals assessed for dementia are in fact suffering from other treatable conditions, such as depression, delirium, side effects from drugs, drug abuse, thyroid dysfunction and vitamin deficiency (Clarfield, 2003). Likewise, many of AD features, such as plaques and tangles, can also be observed in the *post mortem* brain of individuals that do not exhibit dementia (Elobeid et al., 2016). Also, the differences between AD induced cognitive decline and normal aging cognitive changes can be very subtle, making it difficult to establish an early disease diagnosis. The vast majority of AD symptomatic cases (99%) begins after 65 years of age and are referred as late onset or sporadic AD (Holtzman et al., 2011). Although genetic factors may contribute to the risk of developing late onset AD, distinctions can be made between sporadic AD cases and early onset familial AD. Some of the first insights for understanding AD pathogenesis came from the study of familial AD. Familial AD accounts for a small percentage of cases, ∼1%, and is transferred from one generation to the next as an autosomal dominant heritage (Guerreiro et al., 2012). Mutations on the genes encoding for APP (Goate et al., 1991), Presenilin-1 (PS1) (Sherrington et al., 1995) and Presenilin-2 (PS2) (Levy-Lahad et al., 1995; Rogaev et al., 1995) are the cause of most familial cases of AD, with the onset of symptoms occurring between 30 and 60 years of age. Despite the well characterized mutations that lead to familial AD, as mentioned previously, most AD cases are sporadic. Several risk factors have been shown to contribute to non-familial AD, including aging (Ferri et al., 2005), lack of cognitive reserve (Roe et al., 2007), diminished physical activity (Podewils et al., 2005), smoking habit (Anstey et al., 2007), obesity (Lee, 2011) and diabetes (Biessels et al., 2006).

At the cellular level, the major hallmarks of AD are the formation of Aβ plaques and NFTs of hyperphosphorylated tau protein that can be observed specially in the basal forebrain, frontal lobe, hippocampus and cerebral cortex of both human cases and animal models (Duyckaerts et al., 2009; De-Paula et al., 2012; Taipa et al., 2012; Armstrong, 2014; Garin et al., 2015). However, no strict correlation has been found regarding the number of plaques and AD cognitive decline, although synaptic loss is pointed as the major correlate of cognitive impairment (Terry et al., 1991). NFTs are intracellular structures composed of hyperphosphorylated tau protein, a microtubule-associated protein that normally binds tubulin and stabilize microtubules, but that dissociates from tubulin and tends to self-aggregate when it is hyperphosphorylated (Kidd, 1963; Anderton et al., 1987). Oxidative stress, mitochondrial dysfunction, excitotoxicity, neuroinflammation and impaired cholinergic transmission are also key pathological features of the disease (Coyle et al., 1983; Hynd et al., 2004; Moreira et al., 2007; Rojo et al., 2008). As many of these features also overlap with other disorders and occur in healthy individuals, developing accurate biomarkers and models for the disease is a challenging task. The great majority of AD animal models are based on transgenic animals, especially mice. Therefore, the lack of a robust sporadic AD animal model is probably a major pitfall delaying the development of disease-modifying drugs.

Currently, Memantine, an NMDA receptor antagonist that aims to reduce neuronal death triggered by excitotoxicity, and other four different inhibitors of acetylcholinesterase (AChE) that slightly ameliorates AD’s cholinergic deficits, are the only drugs to treat AD patients that is approved by the United States Food and Drug Administration (FDA). Still, none of these drugs delay disease progression. The development of novel molecules targeting different aspects of AD pathology through the use of natural toxins could be a promising approach, taking advantage of these toxins selectivity for different enzymes, channels and subunits (**Table 1**).

**Table 1:** Animal toxins to treat Alzheimer’s disease.

Toxin/Substance	Species of origin	Effects	Experimental model	Reference
Fasciculins	*Dendroaspis* (snake)	Inhibits AChE	Bioinformatics modeling	Mebs, 1989; Harel et al., 1995; Waqar and Batool, 2015
RVV-V	*Daboia russeli russeli* (viper)	Reduces Aβ plaque deposition	SH-SY5Y cell culture	Bhattacharjee and Bhattacharyya, 2013
K-49-P1-20 peptide (isolated from Myotoxin II PLA_2_)	*Bothrops asper* (viper)	Enhances ECE-1 and NEP activities promoting Aβ clearance	HEK293 cell culture	El-Amouri et al., 2008; Smith et al., 2016
SVHRP	*Buthus martensii karsch* (scorpion)	Increases BDNF levels and neurogenesis	*Caenorhabditis elegans* CL4176, CL2006, Cl2355 strains	Wang et al., 2014; Zhang et al., 2016
		Anti-inflammatory		
		Reduces Aβ plaques		
PhTx3-1	*Phoneutria nigriventer* (spider)	Memory improvement	Mice i.c.v. Aβ administration	Gomes et al., 2013; Silva et al., 2016
PhTx4-5-5		Neuroprotective	Hippocampal slices ODLG model	
			Primary corticostriatal neuronal culture	
BVPLA_2_	*Apis mellifera* (Honey bee)	Reduces Aβ plaque deposition	3xTg-AD mice	Ye et al., 2016b
		Cognitive improvement		
		Anti-inflammatory		


Snake-venom-derived toxins have been widely investigated for potential therapeutic applications in AD and other pathologies. These venoms are mainly divided in two groups: neurotoxins and dendrotoxins. The dendrotoxins are isolated from the African mambas (*Dendroaspis* genus), some of which are best known to act as potassium channels blockers (Koh et al., 2006). Displaying much lower toxicity than the other components of *Dendroaspis angusticeps* (green mamba) venom, the fasciculins are known to inhibit AChE activity through binding to a peripheral site of this enzyme, potentiating acetylcholine action and producing generalized muscle fasciculation (Mebs, 1989). Therefore, these toxins could be useful to relief acetylcholine deficits in disorders such as AD. Some efforts have been made in order to determine fasciculin-AChE complex structure and help the design of novel molecules with AChE inhibitory activity. Recent results from bioinformatics modeling revealed similar toxins with AChE potential inhibitory activity, supporting green mamba fasciculins as candidates for the development of AChE inhibitors to be used in AD (Harel et al., 1995; Waqar and Batool, 2015).

The Indian viper *Daboia russelli russelli* produces a venom containing abundant PLA_2_ isoforms, procoagulant enzymes (factor X and V activators) haemorragins, nucleases, proteases, hyaluronidases and several other compounds (Tsai et al., 1996). A recent study has demonstrated that the factor V (RVV-V) component destabilizes Aβ aggregates on *in vitro* cultures of human SH-SY5Y cells incubated with Aβ_42_/Aβ_40_ peptides (Bhattacharjee and Bhattacharyya, 2013). The compound also protected cells against Aβ-induced toxicity. Further investigations discarded proteolysis as a mechanism for this effect, indicating an Aβ aggregate destabilizing activity. Using RVV-V as a template, novel small peptides were synthesized, retaining anti-Aβ-aggregating activity. Moreover, it was reported that one of the peptides has a half-life of 24 h (Bhattacharjee and Bhattacharyya, 2013). Although other previous antiaggregation molecules have been validated in animal models but failed to progress toward clinical use, subsequent studies employing these peptides could hold potential as novel therapeutic tools (Van Dam and De Deyn, 2006).

Metalloproteases have an important role in regulating many physiological processes. ECE1 and NEP are two metalloproteases whose activity degrade Aβ in the brain (Nalivaeva et al., 2012). Different inhibitors of these enzymes have been used several times for different purposes (Lin et al., 2006; Smollich et al., 2007), although stimulators of their activity are much harder to find, except for a few compounds tested *in vitro*, such as green tea polyphenols (Ayoub and Melzig, 2006) and kynurenic acid (Klein et al., 2013). Enhanced ECE1 and NEP activities are thought to have beneficial effects against AD pathology as demonstrated by genetically induced increased expression of ECE1 and NEP on the APP (Choi et al., 2006) and APP/PS1 transgenic mouse models of AD (El-Amouri et al., 2008). A recent study identified the peptide K-49-P1-20 contained in the structure of myotoxin II, a PLA_2_ present in the venom of another viper: *Bothrops asper*. The synthetic peptide was effective to stimulate ECE-1 and NEP activities on the bradykinin-based quenched fluorescent substrate assay, probably via positive allosteric regulation (Smith et al., 2016). Moreover, results from liquid chromatography-mass spectrometry analysis indicates that ECE-1 cleavage of endogenous Aβ40 present in the cerebrospinal fluid obtained from a subject with AD is increased in the presence of K-49-P1-20 peptides, further confirming a potential role for management of Aβ in AD (Smith et al., 2016).

Scorpion toxins are another group of animal toxins with potential therapeutic applications. The *Buthus martensii karsch* (Bmk) scorpion venom has been used in Chinese medicine for the treatment of nervous system disorders for 1000s of years (Wang et al., 2009). Increased neurogenesis, neuron maturation and expression of brain derived neurotrophic factor (BDNF) are reported after treating cultures of neural stem cells with the SVHRP (Wang et al., 2014). Moreover, anti-inflammatory effects of Bmk extracts have also been reported when tested in human chondrocyte and macrophage cultures (Kim et al., 2005). These evidences support a possible role of Bmk venom components such as SVHRP for targeting other neurodegenerative diseases such as AD. Subsequent research provided positive results regarding SVHRP therapeutic use in AD, using the transgenic *Caenorhabditis elegans* Aβ-expressing model (Zhang et al., 2016). Dose-dependent improvements related to reduced oxidative stress, reduced Aβ plaque deposition and Aβ-induced toxicity were observed following SVHRP treatment, indicating that this compound has potential therapeutic effects.

**FIGURE 1:**
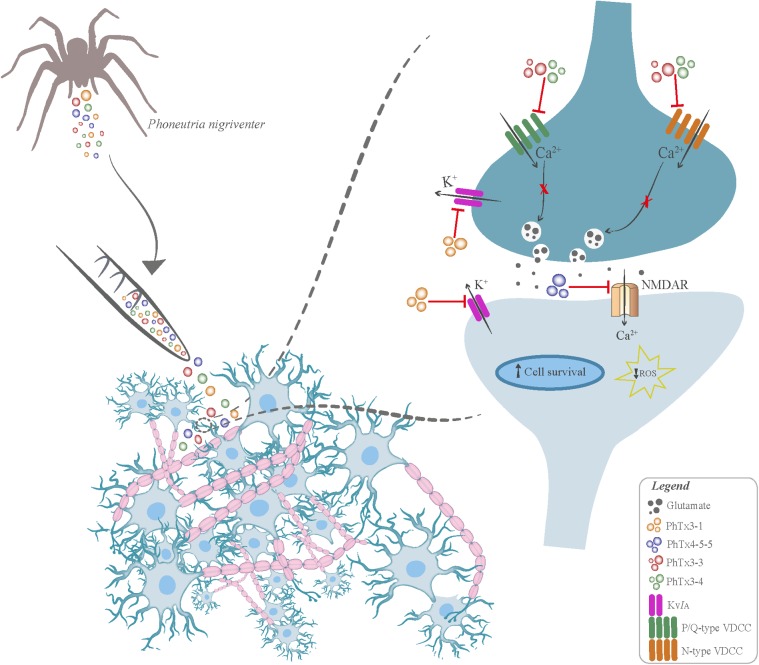
Neuroprotective mechanisms elicited by *Phoneutria nigriventer* venom. The venom of the spider *Phoneutria nigrivente*r contains a mixture of toxins that affect ion channel function, decreasing neuronal cell death and ameliorating neurotransmission alterations. The PhTx3-1 toxin is capable of blocking transient outward K^+^ currents (Kv*I*_A_), improving behavioral parameters associated with memory. The PhTx4-5-5 toxin has neuroprotective activity against glutamate-induced excitotoxicity by blocking NMDA receptors. The PhTx3–3 and PhTx3–4 toxins block N- and P/Q-type voltage-dependent Ca^2+^ channels (VDCC), thus inhibiting Ca^2+^ influx, glutamate release and ROS formation.

The venom obtained from spiders, wasps and bees have also been the subject of many studies. Even though spiders are one of the largest group of venomous animals with approximately 37,000 species, a very small fraction of spider toxins have been characterized so far (Escoubas and Bosmans, 2007). Most of the studies have investigated spider venom toxins for therapeutic applications against cancer, cardiovascular diseases and mostly for analgesic and antinociceptive effects (Pineda et al., 2014). However, there are few studies investigating potential uses of spider toxins in neurodegenerative disorders. Spider venoms contains inorganic ions, free acids and amino acids, glucose, biogenic amines, neurotransmitters and larger protein toxins, and, so far, their main targets are K^+^, Ca^2+^ and Na^+^ channels (Escoubas et al., 2000b). The venom of the Brazilian spider *Phoneutria nigriventer* has been widely studied since the first reports concerning the isolation of different fractions containing several types of toxins. The PhTx3-1 toxin was shown to reduce memory-deficits induced by *i.c.v.* administration of Aβ_25-35_ in Swiss mice, an effect probably attributed to blockage of transient outward potassium currents (**Figure 1**) (Gomes et al., 2013). Furthermore, a recently published study described a toxin also isolated from *Phoneutria nigriventer*, PhKv, that was able to induce antinociception by inhibition of AChE via intrathecal administration in mice (Rigo et al., 2017). Further studies will be important to demonstrate whether PhKv anti-AChE activity could be beneficial in AD. Another *Phoneutria* toxin, PhTx4-5-5, showed neuroprotective activity against Aβ and glutamate-induced excitotoxicity by blockage of NMDA receptors in mice corticostriatal neuronal cultures (**Figure 1**) (Silva et al., 2016). Glutamate excitotoxicity is regarded as an important cell death trigger not only in AD, but also in other neurodegenerative diseases such as HD. Corroborating the hypothesis that *Phoneutria nigriventer* toxins are also valid therapeutic options to treat HD, PhTx4-5-5 was shown to protect corticostriatal neuronal cultures obtained from a mouse model of HD, the BACHD mice (Silva et al., 2016).

The hymenoptera order of insects comprises all species capable of stinging such as bees, wasps and ants. The most studied venoms are those from the *Apis* genus which contain a variety of active components such as peptides (e.g., melittin, apamin), enzymes (e.g., PLA_2_ and hyaluronidases), biogenic amines and others (Son et al., 2007; Matysiak et al., 2011). There are several proposed mechanisms of action for bee venom (BV) and its composing toxins regarding its different therapeutic applications. For instance, BV has anti-inflammatory, antiapoptotic, antioxidative, and antiglutamate induced toxicity actions and is also capable of restoring normal neurotransmitter signaling (Awad et al., 2017). The use of BV to treat dementia-related disorders has been investigated. Lower levels of proinflammatory molecules and increased levels of pERK and BDNF have been observed following BV treatment of an animal model of vascular dementia induced by bilateral common carotid artery occlusion (Cai et al., 2016). LPS intra peritoneal (i.p.) administration induced Aβ accumulation, neuroinflammation and memory loss on male imprinting control region mice (Gu et al., 2015). Administration of BV i.p. inhibited LPS-induced amyloidogenesis, neuroinflammation and memory loss by inhibiting the NF-κB pathway (Gu et al., 2015). More recently, it has been demonstrated that 6 months weekly treatment of 3xTg-AD mice with BV derived PLA_2_ (BVPLA_2_) evoked dramatic reductions of the Aβ deposits in the hippocampus with enhancement of cognitive function (Ye et al., 2016b). Elevated glucose metabolism, reduced microglial activation and CD4^+^ T cell infiltration were also other positive outcomes reported in this study. Furthermore, BVPLA_2_ was at least as effective as Donepezil (a FDA AD approved drug) in ameliorating cognitive and inflammatory processes with lesser adverse effects such as weight loss (Ye et al., 2016b). Indeed, as discussed earlier, PLA_2_ are key enzymes targeting inflammatory associated processes and many authors have also proposed a focus on BVPLA_2_ therapeutic potential (Chung et al., 2015; Lee and Bae, 2016). The expectation is that further research will provide deeper understanding of BV components and mechanisms underlying their therapeutic effects. So far, the observed anti-inflammatory, neuroprotective and antiaggregate activities support the place of BV components as potential tools for treating neurodegeneration.

### Parkinson’s Disease

Parkinson’s disease is the second most prevalent neurodegenerative disease, mainly affecting people over the age of 55. PD is characterized by progressive neurodegeneration in the substantia nigra pars compacta (SNpc), affecting mainly dopaminergic neurons. Moreover, cytoplasmic inclusions called Lewis bodies are observed in the brain of individuals with PD (Lang and Lozano, 1998). Neurodegeneration is the direct cause of PD symptoms, which include bradykinesia, resting tremor and rigidity (Dauer and Przedborski, 2003; George et al., 2009). In addition to these motor alterations, individuals with PD may also exhibit cognitive impairment and psychiatric disturbance (Beitz, 2014). When more than 50% of the SNpc neurons die, the clinical symptoms become evident (Marsden, 1990; Ross et al., 2004). As in the case of AD, PD can be sporadic or hereditary. Notably, familial PD accounts for only 10% of the cases. In addition to genetic predisposition, several other factors contribute to disease pathology, including age-related alterations and environmental toxins (Castrioto et al., 2014). Moreover, only rare cases of familial PD are caused by the mutation of a single gene. Mutations in about 13 PD genes have already been linked to the pathogenesis (Kumar et al., 2012). The genes that are mutated in PD encode proteins that have been shown to play an important role in the disease, including α-synuclein. The presence of toxic aggregated forms of α-synuclein is regarded as a main factor contributing to pathology and having a critical role in microglia-mediated neuroinflammation (Polymeropoulos et al., 1997; Mhyre et al., 2012). In fact, more recently, it has become more evident the central role of chronic inflammation and glial activation as crucial factors inducing the dopaminergic neurodegeneration underlying PD pathogenesis (Loeffler et al., 1994; Cicchetti et al., 2002; Ghosh et al., 2007; McGeer and McGeer, 2008; Hirsch and Hunot, 2009; Qian et al., 2010).

The gold standard therapy for PD patients consists of a combination of carbidopa and levodopa. Levodopa helps to reestablish dopamine levels in the striatum and is effective in reducing motor impairment and disability, whereas carbidopa inhibits the peripheral metabolism of levodopa, thereby allowing its therapeutic concentrations to be achieved in the brain without disabling peripheral adverse effects (Miyasaki et al., 2002; Gazewood et al., 2013). This treatment is usually started when patients begin to experience functional impairment. Although the discovery of levodopa revolutionized PD treatment, after 5 years of therapy, 50% of patients experience motor response complications, associated with involuntary movements called dyskinesias, which are difficult to control and significantly impair quality of life (Miyasaki et al., 2002; Stowe et al., 2008). The alternative treatment consists of dopamine agonists, MAO-B inhibitors and catechol *O*-methyltransferase inhibitors, which improve motor symptoms and functional status, but are less effective than levodopa and also generate an increase in dyskinesias (Stowe et al., 2008; Stathis et al., 2015). Nevertheless, there are no disease-modifying drugs to treat PD patients and most current treatments are symptomatic and none delay dopaminergic neuron degeneration. The ideal approach to treat PD patients should prevent dopaminergic neuronal cell loss and, thereby, slows or even halts disease progression.

**FIGURE 2:**
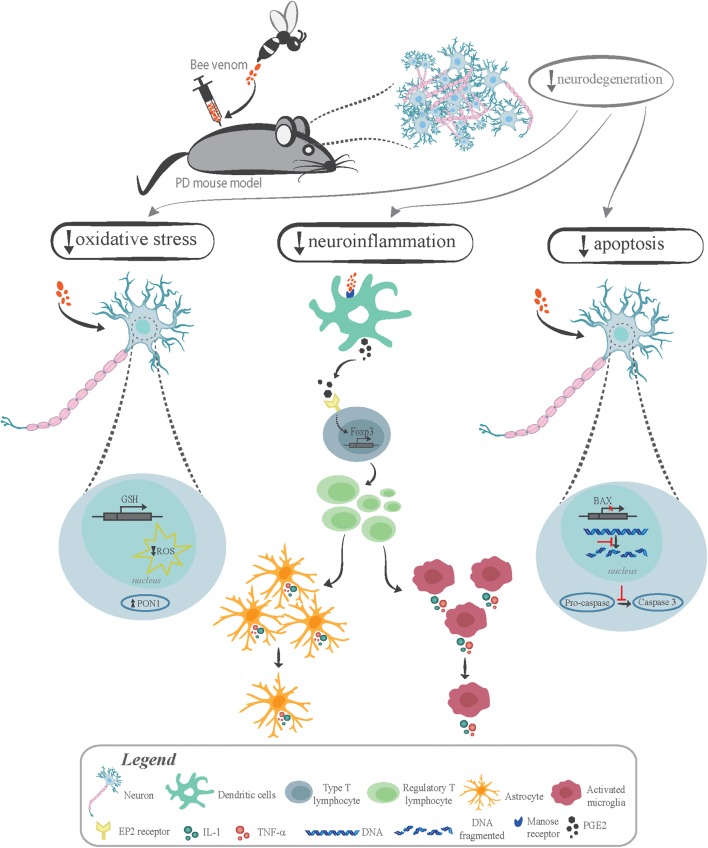
Neuroprotective mechanisms triggered by bee venom in Parkinson’s disease mouse models. The administration of bee venom (BV) or its isolated compounds to different PD mouse models leads to decreased neurodegeneration by reducing oxidative stress, neuroinflammation and apoptosis. BV antioxidant effect is highlighted by its capacity to decrease the levels of reactive oxygen species (ROS), reduce lipid peroxides and restore the antioxidant pool of brain tissue by increasing both glutathione peroxidase (GSH) level and brain PON1 activity. Several studies indicate that BV anti-inflammatory effect is the main mechanism contributing to neuroprotection. BV directly binds to the mannose receptor on dendritic cells and promotes secretion of PGE2, which binds to type T lymphocyte prostaglandin E2 (EP2) receptor, leading to Foxp3 expression. Consequently, it promotes T regulatory lymphocytes (T reg) differentiation, which induces neuroinflammation suppression by decreasing the number and level of activation of both astrocytes and microglia. The shrinkage of inflammatory cells leads to decreased release of proinflammatory factors, such as tumor necrosis factor-α (TNF-α) and interleukin-1 (IL-1). BV also promotes neuroprotection by reducing apoptosis, as it decreases Bax gene expression levels, caspase-3 activation, and DNA fragmentation.

Molecules from BV are currently under investigation as neuroprotective tools to treat PD. It has been shown that chronic release of proinflammatory cytokines by activated astrocytes and microglia exacerbates dopaminergic neuron degeneration in PD. These findings corroborate the hypothesis that inflammatory processes are potential interventional targets in PD and other neurodegenerative diseases (Teismann and Schulz, 2004; Wang et al., 2015). Recent studies suggest that BV could lessen PD-related neuroinflammation (**Figure 2**). One of the first studies indicating BV therapeutic potential in PD showed that BV injection suppresses neuroinflammatory responses in an MPTP-induced mouse model of PD (Kim et al., 2011). MPTP-induced mouse is the most widely used animal model to study this disease. Administration of MPTP to mice leads to dopaminergic cell death in the SNpc and induces a severe and irreversible PD-like syndrome (Przedborski and Vila, 2003; Meredith and Rademacher, 2011). Increased number of activated microglia is also observed in the SNpc and striatum of this mouse model, contributing to secondary dopaminergic PD neuronal cell loss (McGeer et al., 1988, 2003; McGeer and McGeer, 2008). In this study, BV was subcutaneously administered into the acupuncture point in mice (Kim et al., 2011), the most commonly used method for applying BV (Ezzo et al., 2001; Lee et al., 2008; Kim and Jeon, 2014). Acupuncture is a technique used to treat certain illness through stimulation of specific anatomical points using needles, laser or small electrical currents (Lao et al., 2003). A previous study showed that BV acupuncture pretreatment effectively protects dopaminergic neurons against MPTP toxicity by inhibiting Jun activation (Doo et al., 2010). BV reduces microglial activation, which is evidenced by decreased MAC-1 levels, a microglia activation marker, and iNOS expression in the SNpc (Kim et al., 2011). Thus, this study suggests that the dopaminergic neuroprotective effect elicited by BV treatment of MPTP PD model appears to be mainly due to a decrease in neuroinflammation via the suppression of proinflammatory factors, such as cyclooxygenase-2 and PLA_2_, tumor necrosis factor-α (TNF-α) and interleukin-1 (IL-1) (Kim et al., 2011). Consistent with these results, other studies have shown that BV treatment reduces microglial activation and the infiltration of CD4 T effector cells into the SNpc (Chung et al., 2012, 2015; Ye et al., 2016a). Additionally, BV treatment significantly increases the proportion of regulatory T cells (T reg) *in vivo* and *in vitro* and T reg depletion abrogates the neuroprotective effects of BV (Chung et al., 2012). T regs play an important role in the regulation of the immune response of peripheral CD4 T cells, thus being a crucial step in the maintenance of tolerance in healthy conditions (Sakaguchi et al., 2008). In addition, adoptive transfer of T regs to MPTP-treated mice prevents PD-related neuronal degeneration through attenuation of microglial activation and neuroinflammatory responses (Reynolds et al., 2007). Therefore, these studies suggest that the neuroprotective effects of BV treatment are mediated in part by the modulation of the adaptive immune response by increasing the proportion of functional T regs (Chung et al., 2012). Subsequently, the same research group demonstrated that BVPLA_2_, the major BV compound, is capable of inducing T reg expansion, promoting survival of dopaminergic neurons. The results also showed that BVPLA_2_ directly binds to mannose receptor on dendritic cells and, consequently, promotes secretion of PGE2, which results in Treg differentiation in the MPTP model of PD (Chung et al., 2015) (**Figure 2**).

BVPLA_2_ has a wide variety of pharmacological properties, including anti-HIV activity, myotoxicity and neurite outgrowth induction (Fenard et al., 2001; Nakashima et al., 2004). Moreover, in a human A53T α-Syn mutant transgenic mice (A53T Tg) the expression of α-synuclein was reduced, as well as microgliosis in spinal cord and the M1/M2 ratio through the treatment with BVPLA_2_ (Ye et al., 2016a). It has been shown that the phenotype of activated microglia (M1/M2) controls the repair and regeneration response following nerve injury (Kigerl et al., 2009; David and Kroner, 2011). Several studies classify microglia into two different polarizations: M1 and M2. The first is related to proinflammatory action and release of cytokines such as TNF-α. On the other hand, M2 is important for homeostasis, with anti-inflammatory action and producing cytokines such as IL-10 (Orihuela et al., 2016; Tang and Le, 2016). However, several other studies show opposite results and do not support this notion that M1 is pro and M2 is anti-inflammatory (Gautier et al., 2012; Miller et al., 2012). At the behavioral level, BV treatment ameliorates motor coordination and balance of A53T Tg mice in a modified pole test (Ye et al., 2016a). Previous studies have also shown the beneficial actions of BV treatment on the pathological functioning of the circuits underlying motor PD symptoms (Maurice et al., 2015; Kim et al., 2016). Therefore, these findings suggest that BVPLA_2_ is a relevant pharmacological tool in PD.

Another specific component of BV, apamin, was also studied in isolation. Previous studies reported that the peptide apamin protects cultured mesencephalic dopaminergic neurons (Salthun-Lassalle et al., 2004; Toulorge et al., 2011). Employing the MPTP-induced PD model, which was exposed to either BV or apamin treatment via intraperitoneal injections, it was possible to observe that the BV protective effect is not restricted to acupoint stimulation. High doses of the BV peptide apamin lead to the same level of protection of the cellular bodies of dopaminergic neurons and also diminish inflammatory cytokines, which can be deleterious. However, this protection cannot be seen at the axonal level when only apamin is used, whereas BV grants safety of this site, suggesting that other molecules might be involved in the axonal protection phenomena (Alvarez-Fischer et al., 2013).

Interestingly, a study compared the neuroprotective effect of BV and Pramipexole on the progressive neuronal damage and motor dysfunctions observed in a subchronic MPTP mouse model of PD. Pramipexole is a potent and selective D3 dopamine receptor agonist that is clinically important for managing early stage PD (Mierau and Schingnitz, 1992; Bennett and Piercey, 1999). Furthermore, Pramipexole has been shown to exert a neuroprotective effect against MPTP-induced damage to the nigrostriatal dopamine system in mice (Anderson et al., 2001). Significant astrocytic activation is observed following MPTP subchronic administration and both BV and Pramipexole effectively reduce activation of astrocytes in SNpc, decreasing death of dopaminergic neurons. Moreover, both BV and Pramipexole ameliorate motor deficits, whereas, in primary cultured astrocytes, only BV reduces MPP+-induced astroglial activation. Taken together, these observations suggest that both BV and Pramipexole effectively reduce PD-associated pathologies and thus the neuroprotective effects of BV are associated with reduced astrocytic activation (Kim et al., 2016).

The neuroprotective effect of BV acupuncture therapy was also evaluated in neurodegeneration induced by rotenone. Rotenone, a natural insecticide, is extremely lipophilic and easily crosses the blood-brain barrier (BBB) (Talpade et al., 2000). A Parkinsonism rat model can be created by chronic exposure to rotenone leading to motor deficits and dopaminergic neuronal loss (Deng et al., 2010). This study further indicates that BV therapy has a neuroprotective effect that is mediated through inhibition of neuroinflammation, oxidative stress and apoptosis. BV powerful antioxidant effect is highlighted by its capacity to reduce lipid peroxides and restore the antioxidant pool of brain tissue by increasing both glutathione peroxidase level and brain paraoxonase-1 (PON1) activity, preventing neuronal injury. BV also decreases Bax gene expression levels and suppresses apoptotic pathways, which is further demonstrated by decreased DNA fragmentation and suppressed caspase-3 activation induced by rotenone (Khalil et al., 2015) (**Figure 2**).

Based on all these findings, clinical studies were performed to test whether BV could provide an effective option to treat neurodegenerative diseases, including PD (Mirshafiey, 2007). One of these studies explored the benefits of both acupuncture and bee venom acupuncture as adjuvant therapies for idiopathic PD (Cho et al., 2012). Forty-three adults with idiopathic Parkinson’s disease were recruited and assessed using the Unified Parkinson’s Disease Rating Scale, the Parkinson’s Disease Quality of Life Questionnaire, the Beck Depression Inventory, the Berg Balance Scale, and the time and number of steps required to walk 30 m. Subjects were stimulated twice a week for 8 weeks. In this pilot study, both acupuncture and bee venom acupuncture showed promising results as adjuvant therapies for Parkinson’s disease (Cho et al., 2012). In another monocentric double-blinded, randomized controlled pilot study, 40 PD cases were evaluated regarding the potential disease-modifying effects of BV monthly injections. The results obtained in this study indicate that BV treatment does not have any clear effect on PD symptomatology, but highlights the importance of further studies using higher doses and higher administration frequency of BV (Hartmann et al., 2016).

In addition to BV, some other animal venoms are also being investigated as potential therapeutic strategies for PD treatment. For instance, it has been shown that the venom of the Chinese scorpion Bmk protects dopaminergic neurons in the SNpc and improves the related behavior deficits in PD models (Xu et al., 2015). At early stage PD, the scorpion venom peptide, SVHRP, protects against oxidative stress. The antioxidant actions and the protection of mitochondria by SVHRP were studied by using the 6-OHDA rat model for early PD (Yin et al., 2014). 6-OHDA is an oxidative stress neurotoxin that increases oxidative damage and decreases antioxidant ability in midbrain (Kirik et al., 1998). SVHRP was found to be successful in reversing the abnormal activities of MAO-B, SOD, and MDA in the mitochondria of neurons in the midbrain, and in improving the antioxidant ability in an early stage PD rat model (Yin et al., 2014). In addition, SVHRP preserves the function of axons and promotes up-regulation of Bax and down-regulation of Bcl-2 (Wang and Qin, 2010; Yin et al., 2014; Xu et al., 2015).

Snake venom has also been studied as a therapeutic tool to treat PD. The neuroprotective activity and ability to induce neuritogenesis of a peptide isolated from the *Bothrops atrox* venom fraction Ba-IV was investigated using PC12 cells treated with the dopaminergic neurotoxin MPP+. This peptide, which had its sequence identified as Glutamic acid–Valine–Tryptophan, is able to significantly reduce cell death and this protective effect is associated with decreased activity of two apoptotic proteases, caspase-9 and caspase-3. In addition, neurites outgrowth was observed in this Parkinson’s cellular model after peptide treatment, indicating that the underlying mechanism of protection might include a neurotrophic effect (Martins et al., 2015).

Considering all these evidences, it is clear the importance and the great potential of these animal venoms as prospective pharmacological tools to treat PD (**Table 2**).

**Table 2:** Animal toxins to treat Parkinson’s disease.

Toxin/Substance	Species of origin	Effects	Experimental model	Reference
Bee Venom	*Apis mellifera* (Honey bee)	Anti-inflammatory	MPTP mouse model	Doo et al., 2010;
		Reduces microglial activation and CD4+	A53T Tg mice	Kim et al., 2011;
		T cells infiltration	Rotenone-induced parkinsonism model	Chung et al., 2012, 2015;
		Improves motor coordination and balance		Khalil et al., 2015
		Reduces apoptotic markers		Ye et al., 2016a
		Reduces oxidative stress		
BVPLA_2_	*Apis mellifera* (Honey bee)	Promotes T reg cells expansion	A53T Tg mice	Chung et al., 2015;
		Neuroprotective for dopaminergic neurons		Ye et al., 2016a;
		Reduces α-synuclein expression		
		Reduces activation of microglia and macrophages		Kim et al., 2016
Apamin	*Apis mellifera* (Honey bee)	Neuroprotective	Rat mesencephalic dopaminergic neuronal culture	Salthun-Lassalle et al., 2004;
		Anti-inflammatory	MPTP PD model	Toulorge et al., 2011;
		Improves motor function		Alvarez-Fischer et al., 2013
Bee Venom	*Apis mellifera* (Honey bee)	Improves scores of Unified PD Rating Scale, Berg Balance Scale and 30 m walking time	Clinical Trial	Cho et al., 2012	Hartmann et al., 2016
SVHRP	*Buthus martensii karsch* (scorpion)	Antioxidant	6-OHDA rat model	Yin et al., 2014;
		Antiapoptotic		Xu et al., 2015
		Neuroprotective for SNpc dopaminergic neurons		
		Behavior improvements		
Tripeptide (Glutamic acid–Valine–Tryptophan)	*Bothrops atrox* (snake)	Antiapototic	PC12 MPP+ *in vitro* model	Martins et al., 2015
		Enhances neurite outgrowth		


### Brain Ischemia

Ischemia is caused by deficient blood supply to tissues due to obstruction of the arterial flow. Most ischemic episodes are caused by thromboembolic or atherothrombotic vaso-occlusive disease and the major risk factors are age, hereditary factors, tobacco smoking, hyperlipidemia, hypertension, physical inactivity, obesity, and diabetes mellitus (Kalogeris et al., 2012). Clinically, the most significant event is brain ischemia, as neuronal cells are more sensitive to reduction in blood supply due to their intense metabolic activity (Kristian, 2004). Brain ischemia is followed by irreversible damage already detectable at less than 20 min after the ischemic event and, in most cases, resulting in severe brain damage, which is the leading cause of death and long-term disability worldwide (Ordy et al., 1993). The primary consequence of brain ischemia is a reduction in energy substrates, including glucose and oxygen. Consequently, ATP synthesis through glycolysis and oxidative phosphorylation slows or even stops, promoting a rapid decline in cellular ATP levels (Silver et al., 1997). Furthermore, the interruption of oxidative phosphorylation causes an increase in the release of ROS (Abramov et al., 2007). Finally, when respiration is inhibited but glycolysis persists, protons and lactate generated during glycolysis accumulate, causing rapid intracellular acidification (Silver et al., 1997). Thus, ischemia invariably causes depletion of cellular ATP, intracellular acidification and generation of ROS, triggering a sustained rise of glutamate extracellular concentration (Phillis et al., 1996). This cascade of events culminates in a large elevation of intracellular Ca^2+^ in neurons and astrocytes, which triggers the death of neurons (Schubert et al., 1994; Rossi et al., 2007).

Ischemia treatment consists in the use of thrombolytics, aiming to lyse the arterial thrombus to restore blood flow to poorly perfused areas (thrombolysis) and to reduce the intrinsic vulnerability of the brain tissue to ischemia. However, the ischemia-treatment time interval determines therapy success. The exposure to a short but severe ischemia delays cell death and affects only a reduced number of neurons, whereas when ischemia last for a long period of time, broader and more rapid cellular destruction is observed, resulting in either focal or global ischemia, with the last leading to death in most cases (Lipton, 1999). Although thrombolytics help the prognosis, treatment is still far from satisfactory, mainly due to the limited therapeutic time window of approximately 3 h and the potential side effect of intracranial hemorrhage. To date, no neuroprotective drug has proven to be effective in phase III clinical trial studies. In addition, the heterogeneity of the disease makes it difficult to draw conclusions from the different studies. Therefore, the search for efficient drugs is an urgent unmet need.

Compelling evidences indicate that increased extracellular glutamate levels and exacerbated cytosolic Ca^2+^ overload are the main causes of neuronal injury during cerebral ischemia (Choi, 1994; Wheeler et al., 1994; Choi, 1995). Thus, drugs able to block these two events constitute potentially effective pharmacological strategies against the harmful consequences of ischemia. Studies investigating several animal venoms revealed a large amount of toxins capable of specifically blocking different Ca^2+^ channels types (Uchitel, 1997). Among the venoms, the ω-conotoxins from the venom of the fish-eating marine snail *Conus magus* deserve especial attention. One of the ω-conotoxins is MVIIC, which is comprised of 26 amino acids and is a member of the Ca^2+^ channels blockers toxin family (Hillyard et al., 1992; Liu et al., 1996). *In vitro* studies evaluating cerebral and spinal cord ischemia demonstrated that MVIIC significantly reduces Ca^2+^ influx and attenuates the release of glutamate (Liu et al., 1996; Imaizumi et al., 1999). Based on these results, an *in vivo* study using a rat model of spinal cord ischemia confirmed that MVIIC is capable of decreasing glutamate release and Ca^2+^ influx into the cell (Oliveira et al., 2014). Moreover, MVIIC treatment also preserves neuronal integrity, reduces cell death and hemorrhagic process, and leads to enhanced performance in behavioral tests (Oliveira et al., 2014).

MVIIA, which is an N-type Ca^2+^ channel blocker, is another ω-conotoxin that has been extensively studied for its neuroprotective properties. Notably, the development of a synthetic version of MVIIA, the SNX-111, facilitated further studies (Olivera et al., 1987). The first studies showed that SNX-111 is effective in preventing neuronal damage after transient global ischemia in rat even when administered up to 24 h after the ischemic insult (Valentino et al., 1993). Using a transient focal ischemia rat model, which was subject to 2 h MCA occlusion, it was demonstrated that SNX-111 drastically ameliorates brain damage and reduces infarct size (Zhao et al., 1994). Also employing a focal cerebral ischemia rat model, it was shown that SNX-111, administered intravenously at 5 mg/kg/h from 20 min prior to occlusion until 2 h post occlusion, significantly reduces extracellular glutamate level through inhibition of its presynaptic release. This result was associated with a reduction in the cortical size infarction and neuroprotection (Takizawa et al., 1995). Similar results were observed in a focal cerebral ischemia rabbit model and in a global ischemia rat model (Buchan et al., 1994; Perez-Pinzon et al., 1997). Taking into account all these studies, pharmacokinetic studies were conducted in rats and cynomologus monkeys to determine SNX-111 disposition following 24 h of continuous, constant-rate intravenous infusion (Bowersox et al., 1997). However, despite all these positive results *in vitro* and *in vivo*, when clinical trials were performed to evaluate ZNX-111 effect on ischemia-induced brain injury and chronic pain, positive results were only obtained in the case of chronic pain. Based on these results, the FDA approved the use of SNX-111, which is also known as Ziconotide (Prialt^®^; Elan Pharmaceuticals, Inc.), for the management of severe chronic pain in patients whom intrathecal therapy is authorized, and who are intolerant of or refractory to other treatments (Heading, 2001; McGivern, 2007; Smith and Deer, 2009).

Besides MVIIC and MVIIA, another ω-conopeptide called GVIA, from snail *Conus geographus*, has also been evaluated for its neuroprotective properties. *In vitro* experiments showed that GVIA inhibits excessive release of glutamate during ischemia by blocking N-type Ca^2+^ channel and that this inhibition generates significant protective effect in neurons (Madden et al., 1990; Wang et al., 1992). Regardless of these neuroprotective effects, GVIA has not been extensively studied because its characteristic irreversible binding to the N-type VGCCs limits its clinical potential utility (Wang et al., 1992).

Toxins contained in spider venom have also been shown to inhibit ion channels. For instance, the spider *Phoneutria nigriventer* venom contains a mixture of toxins that affect ion channels and that have been investigated for the treatment of neurodegenerative processes (**Figure 1**). The *Phoneutria nigriventer* venom fraction PhTx3 contains a broad-spectrum neuronal Ca^2+^ channel blocker that also inhibits glutamate uptake (Reis et al., 1999). The neuroprotective effects of PhTx3, GVIA and MVIIC were evaluated employing hippocampal slices and mouse cholinergic septal neuronal cell line (SN56 cells), which were subjected to ischemia by oxygen deprivation and low glucose insult. The results from this study indicate that PhTx3 completely rescues neuronal cell death, although both snail toxins afford only partial cell protection (Pinheiro et al., 2006). After this study, the neuroprotective potential of some of the individual components present in the PhTx3 fraction were evaluated. The results demonstrated that the PhTx3 components, PhTx3–3 and PhTx3–4, inhibit Ca^2+^ influx, glutamate release, and exocytosis in nerve endings (Guatimosim et al., 1997; Miranda et al., 1998; Reis et al., 1999). Next, the *in vitro* action of PhTx-3-3 and PhTx3-4 on brain injury induced by oxygen deprivation and low glucose insult was investigated using slices of rat hippocampus. In addition to confirm that PhTx-3-3 and PhTx3-4 inhibit the increase of glutamate release, this study showed that these toxins prevent neuronal cell death and rescue the neurotransmission alterations observed in hippocampus CA1 when applied before and after the onset of ischemia (**Figure 1**) (Pinheiro et al., 2009).

Studies have demonstrated that, apart from excitotoxicity, activation of the Ca^2+^ permeable ASICs is largely responsible for the development of acidosis-mediated, glutamate-independent ischemic brain injury (Astrup et al., 1977; Xiong et al., 2004). Postsynaptic ASIC1a is the dominant ASIC subtype in both human and rodent brains and, therefore, its modulation is a potential therapeutic target for ischemia (Li et al., 2010). One of the most potent blockers of ASIC1a is PcTX, which is obtained from the venom of the South American tarantula, *Psalmopoeus cambridgei.* Using a rat model of transient focal ischemia, the first studies investigating this venom confirmed the crucial role of ASIC1a in the pathogenesis of ischemia and the neuroprotective effect of PcTX (Pignataro et al., 2007). It was found that PcTX is able to reduce infarct volume, even if administered after permanent occlusion of the middle cerebral artery, suggesting that ASIC can be activated in the absence of reperfusion. Additionally the intranasal administration of PcTX is nearly as effective as PcTX i.c.v. administration (Xiong et al., 2004; Pignataro et al., 2007). Another study confirmed the beneficial action of PcTX in a newborn piglet model of asphyxia-induced cardiac arrest. Treatment with PcTX reduces striatonigral and striatopallidal neuronal injury, attenuates increased protein kinase A-dependent phosphorylation of DARPP-32 and NMDA receptor NR1 subunit and decreases nitrative and oxidative damage to proteins (Yang Z.J. et al., 2011). The aforementioned studies used whole venom from the spider *Psalmopoeus cambridgei.* However, it has already been demonstrated that PcTx1, a 40-residue peptide that constitutes a very minor proportion (∼0.4%) of this venom, is the most selective blocker of ASIC1a (Escoubas et al., 2000a). Moreover, recombinant PcTx1 and PcTx1a are equipotent regarding neuroprotection in a conscious hypertensive rat model of transient MCA occlusion (Saez et al., 2011). In addition, animals submitted to conscious stroke that were treated with PcTx1 display intact neuronal architecture, increased number of neurons, and reduced number of caspase-3 positive cells, indicating that this peptide is efficient to prevent apoptosis and foster neuronal survival (McCarthy et al., 2015).

Several other animal toxins have also been shown to be capable of reducing ischemic injury. HWTX-I, an N-type Ca^2+^ channel blocker, is the most abundant toxic component in the crude venom of the Chinese bird spider *Ornithoctonus huwena* (Liang et al., 1993). Using a rat model of global cerebral ischemia-reperfusion injury, it was demonstrated that HWTX-I modulates the expression and activity of many apoptosis pathway components, including SOD, glutathione peroxidase, Fas, TNFα, caspase-8 and caspase-3 (Wang et al., 2007). Snakes, such as *Bothrops atrox* and *Bothrops brazili*, have in their venoms serine proteinases capable of promoting angiogenesis through the PI3K/Akt pathway (Bhat et al., 2016). Also by activating the PI3K/Akt pathway, rLj-RGD3, a recombinant toxin from the salivary gland of the *Lampetra japonica* fish, protects against cerebral ischemia/reperfusion damage (Lu et al., 2016). rLj-RGD3 significantly ameliorates pathological changes in the brain and inhibits neuronal apoptosis by increasing the expression of FAK, p-FAK and Bcl-2 proteins and decreasing the expression of caspase-3 (Lu et al., 2016).

Therefore, many studies have searched for new and effective treatments for ischemia using animal toxins as pharmacological tools (**Table 3**). Ischemia involves a complex pathogenic cascade of events, which includes energy depletion, excitotoxicity, acidosis, and peri-infarct depolarization, and thus the ideal treatment might need to make use of combined therapies maybe employing two or more toxins.

**Table 3:** Animal toxins to treat brain ischemia.

Toxin/Substance	Species of origin	Effects	Experimental model	Reference
ω-conotoxin MVIIC	*Conus magus* (snail)	Neuroprotective	Spinal cord neuronal cell culture	Liu et al., 1996;
		Reduces Ca^2+^ influx and glutamate release	Spinal cord ischemia rat model	Imaizumi et al., 1999;
		Reduces hemorrhage		Oliveira et al., 2014
		Improves performance in behavioral tests		
ω-conotoxin MVIIA/SNX-111	*Conus magus* (snail)	Neuroprotective	Rat cerebral transient focal ischemia model	Valentino et al., 1993;
		Reduce neuronal damage	Rabbit cerebral focal ischemia model	Zhao et al., 1994;
		Reduce infarct size	Rat global ischemia model	Takizawa et al., 1995;
		Reduce glutamate release		Buchan et al., 1994;
				Perez-Pinzon et al., 1997
ω-conotoxin GVIA	*Conus geographus* (snail)	Neuroprotective	Rat cortical neuronal culture hypoxia model	Madden et al., 1990;
		Reduces glutamate release	Rabbit spinal cord transient ischemia model	Wang et al., 1992
PhTx3 (Tx3-3 and Tx3-4)	*Phoneutria nigriventer* (spider)	Neuroprotective	Rat hippocampal slices ODLG model	Pinheiro et al., 2006;
		Reduce Ca^2+^ and glutamate release	SN56 cells ODLG model	Guatimosim et al., 1997;
		Restore normal neurotransmission		Miranda et al., 1998;
				Reis et al., 1999
Psalmotoxin-1 (PcTX)	*Psalmopeous cambridgei* (spider)	Reduces infarct volume, neuronal damage and oxidative stress	Rat cerebral transient focal ischemia model	Escoubas et al., 2000a;
		Reduces excessive NMDA NR1 subunit and DARPP-32 phosphorylation	Piglet model of asphyxia-induced cardiac arrest	Xiong et al., 2004;
		Antiapoptotic	Hypertensive rat model of transient MCA occlusion	Pignataro et al., 2007;
				Saez et al., 2011;
				Yang Z.J. et al., 2011;
				McCarthy et al., 2015
Huwentoxin-I (HWTX-I)	*Ornithoctonus huwena* (spider)	Antiapoptotic	Rat global ischemia-reperfusion model	Wang et al., 2007
Serine proteases	*Bothrops asper* and *brazili* (snakes)	Promote angiogenesis	Endothelial, Fibroblast and HEK293 cell culture	Bhat et al., 2016
			Agarose plug transplantation assay	
rLj-RG	*Lampetra japonica* (fish)	Neuroprotective	Antiapoptotic	Rat middle cerebral artery occlusion model	Lu et al., 2016


### Glaucoma

Degeneration of RGCs is a key feature of major ophthalmologic conditions such as glaucoma, diabetic retinopathy and retinal ischemia. The second most common cause of blindness is glaucoma. The disease is characterized by degeneration of RGCs and atrophy of intracranial optic nerves, lateral geniculate nucleus and visual cortex (Gupta et al., 2006). Glaucoma shares several common mechanisms with other neurodegenerative diseases, including oxidative stress, impaired axonal transport, neuroinflammation, excitotoxicity and even deposition of Aβ, α-synuclein and phosphorylated tau in the retina (Gauthier and Liu, 2016; Ramirez et al., 2017). Thus, although glaucoma was originally regarded as an eye disease, more recently, it has been suggested that it could be considered a CNS degenerative disease (Weber et al., 2000; Yucel et al., 2003; Gupta et al., 2006). Elevated intra-ocular-pressure is a hallmark of the disease and also the only modifiable factor for therapeutically targeting the pathology (Gauthier and Liu, 2016). The exact mechanisms underlying disease progression are still largely unknown.

Recent studies have demonstrated that spider venom compounds hold potential to treat glaucoma (**Table 4**). For instance, it has been shown that components of the Brazilian spider *Parawixia bistriata* venom are neuroprotective. Using a rat glaucoma model based on ischemia/reperfusion of retina, it was shown that the compound called FrPbAII promoted neuroprotection of the retinal cell layers and was also capable of crossing the BBB (Beleboni et al., 2006). Parawixin 1, another compound purified from the venom of *Parawixia bistriata*, similarly to FrPbAII, has neuroprotective effects demonstrated on retina ischemia/reperfusion glaucoma models (Fachim et al., 2015). Also from *Parawixia bistriata* venom, PbTx1.2.3, has protected neurons from degeneration in the ischemia/reperfusion retina model, probably through anti excitotoxic activity (Fontana et al., 2003). These results strongly suggest that *Parawixia bistriata* venom components are potential therapeutic tools.

**Table 4:** Animal toxins to treat glaucoma.

Toxin/Substance	Species of origin	Effects	Experimental model	Reference
FrPbAII	*Parawixia bistriata* (spider)	Neuroprotective	Rat retinal cells culture ischemia/reperfusion model	Fontana et al., 2003; Beleboni et al., 2006; Fachim et al., 2015
Parawixin 1				
PbTx1.2.3				
PhTx3-3	*Phoneutria nigriventer* (spider)	Neuroprotective	Rat retinal cells culture ODLG model	Agostini et al., 2011; Binda et al., 2016
PhTx3-4		Reduce ROS, oxidative stress and degradative enzymes	*In vivo* NMDA intravitreal administration in rats	


The venom of *Phoneutria nigriventer* has also been studied for retina neuroprotection. Excessive influx of Ca^2+^ through VGCCs triggers the activation of degradative enzymes, increases the levels of ROS and free radicals and promotes oxidative stress in the cell (Siesjo, 1992). It is therefore easy to assume that blockage of VGCCs could hold therapeutic potential. Indeed, PhTx3-3 and PhTx3-4 toxins purified from *Phoneutria nigriventer* venom, blockers of N-P/Q Ca^2+^ channels, protect rat retinal slices submitted to the oxygen deprivation and low glucose (ODLG) ischemic insult model (**Figure 1**) (Agostini et al., 2011). Subsequent research demonstrated that PhTx3-3 has an *in vivo* neuroprotective effect over rat retinas challenged with NMDA induced injury. Results showed reduced glutamate release as well as reduced levels of ROS, free radicals, oxidative stress and degradative enzymes following pretreatment with PhTx3-3 (Binda et al., 2016). These results suggest a role for *Phoneutria nigriventer* venom toxins as potential therapeutic agents for managing neurodegenerative retinopathies, encouraging further studies.

### Amyotrophic Lateral Sclerosis

Amyotrophic lateral sclerosis is a neurodegenerative disease characterized by progressive degeneration of upper and lower motoneurons and that is ultimately fatal (Rowland and Shneider, 2001). The sporadic form of the disease accounts for most of the cases (∼90%), indicating that no obvious genetic component is responsible for triggering the disease. Meanwhile, the remaining 10% of the cases are attributed to genetic mutations constituting the familial cases of ALS (Greenway et al., 2006; Abhinav et al., 2007). The major symptoms are general muscle spasticity, fasciculation, atrophy and paralysis, leading to death by respiratory failure in 3–5 years from symptoms onset (Walling, 1999). Some studies suggest an association of increased risk to develop ALS and cigarette smoke (Weisskopf et al., 2009), exposure to chemical and metal contaminants (Yu et al., 2014; Roberts et al., 2016), and exposure to radiation and electromagnetic fields (Phillips et al., 1998). Athletes have a higher risk to develop the disease, although there are controversial results regarding physical activity and ALS incidence (Beghi et al., 2010; Huisman et al., 2011). In that regard, several genes previously recognized as ALS risk factors are also related to exercise, which could explain these controversial data (Chen et al., 2004). Riluzole is the only currently available pharmacotherapy to treat ALS patients, extending life expectancy in about 3 months. Although Riluzole mechanism of action is not completely understood, it has been shown that it probably involves inhibition of ionic channels such as Na^+^, K^+^, and Ca^2+^ channels (Bensimon et al., 1994; Bellingham, 2011).

Within the familial cases of ALS, mutations on the SOD1 gene are the most common (Dangoumau et al., 2014; Zarei et al., 2015). Treatment of symptomatic SOD1 mutant mice (hSOD1^G93A^) with BV promotes extended survival, improved motor function, reduced microglial activation and improved mitochondrial integrity (Yang et al., 2010). Further research demonstrated that melittin, isolated from BV, also decreases pathological inflammation, improves motor function and reduces neuronal cell death and α-synuclein misfolding (Yang E.J. et al., 2011). Other studies report the efficacy of BV in reducing expression of inflammatory mediators in the lungs, liver, spleen and kidneys of hSOD1^G93A^ mice. Moreover, these effects might be modulated by the selection of specific acupuncture points (Lee et al., 2014, 2015). Another *in vitro* study used NSC43 motor neuronal cells transfected with either WT or a GFP-hSOD1^G58R^ construct to assess BV treatment effects (Kim et al., 2013). GFP-hSOD1^G58R^ overexpression induced formation of SOD1 inclusions and inhibited proteasome activity. Both effects were reverted by BV treatment, although no autophagic pathway was activated (Kim et al., 2013).

Finally, some studies have demonstrated that the SSM venom has a positive effect on various diseases, including cancer, stroke and epilepsy (Gomes et al., 1983; Cai et al., 2013). However, in most studies, SSM extract and not isolated toxins was tested. SSM extract was also investigated in ALS pathology. Treatment with SSM extract significantly protected hSOD1^G93A^ mice lumbar spinal cord cells from neurodegeneration (Cai et al., 2013). Thus, compounds found in BV and SSM venom are potential therapeutic tools to treat ALS (**Table 5**).

**Table 5:** Animal toxins to treat amyotrophic lateral sclerosis.

Toxin/Substance	Species of origin	Effects	Experimental model	Reference
Bee venom	*Apis mellifera* (Honey bee)	Increase survival	hSOD1^G93A^ mice	Yang et al., 2010;
Mellitin		Improve motor function	hSOD1^G58R^ mice	Yang E.J. et al., 2011;
		Anti-inflammatory		Kim et al., 2013
		Neuroprotective		
		Reduce α-synuclein misfolding		
SSM venom extract	*Scolopendra subspinipes mutilans* (centipede)	Neuroprotective	hSOD1^G93A^ mice	Cai et al., 2013


### Multiple Sclerosis

In contrast to the other neurodegenerative diseases, MS is an autoimmune disease, remarkably characterized by pathologic inflammation and increased immunologic activity (Katz Sand, 2015; Pawate and Bagnato, 2015). MS is often described as a T cell-mediated disease, as a common MS feature is that Th-17 CD4^+^ cells cross the BBB and cause neuronal damage such as axon demyelination and neuronal cell death (Kebir et al., 2007). Th-17 lymphocyte cells secrete interleukin-17, an inflammatory mediator that is inhibited by IFNβ. Indeed, the approval of therapeutic administration of IFNβ to MS patients in 1993 was a landmark for disease treatment (Paty and Li, 1993). Men are twice as likely to be affected by the disease and the main symptoms are gait problems, visual impairment, fatigue, sexual and bladder dysfunctions (smooth muscle dysfunctions), dementia and others (Halbreich, 1993). MS pathogenesis is complex and can be classified according to disease progression. A recent review has summarized the current classification of the five MS subtypes: Relapse remitting MS, clinically isolated syndrome, radiologic isolated syndrome, primary progressive MS and secondary progressive MS (Lublin, 2014). The majority of MS cases (∼80%) are relapse remitting, which means that acute periods of disease exacerbation are followed by periods of complete recover and stability.

All MS available treatments are aimed toward reducing relapse frequency and severity, avoid permanent disability and delay or even prevent progression to secondary progressive MS, although so far no curative drug has been developed (Ontaneda et al., 2012; Kamm et al., 2014). Approved drugs are glatiramer acetate, teriflunomide, natalizumab, fingolimod, mitoxantrone, and different isoforms of IFNβ. Corticosteroids have also long been applied for treating the inflammatory and immunomediated character of the disease (Ontaneda et al., 2012; Kamm et al., 2014).

Fortunately, current pharmacotherapy for MS is constantly expanding, even though only modest improvements are achieved through these drugs, which implicates in an ongoing need for novel therapeutic options. Venom based therapy in MS has been explored in several studies showing some positive evidences (**Table 6**). ShK, a sea anemone (*Stichodactyla helianthus*) toxin blocker of Kv1.3 channels, which are crucial for activated T lymphocytes action, has shown beneficial effects on autoimmune encephalomyelitis, a MS rat model (Norton et al., 2004). Scorpion venom components have long been described as therapeutic options to treat the disease. Impaired nerve conduction was reversed by scorpion venom treatment (Adam et al., 1966; Bostock et al., 1978). Furthermore, a case report describes that a 43 year old man affected by MS for 3 years was bitten by a scorpion and several improvements in MS symptoms took place until the man was completely asymptomatic for the following 2 months (Breland and Currier, 1983). Kaliotoxin, isolated from scorpion venom, is also a highly selective blocker of Kv1.3 channels. Positive results in the experimental encephalomyelitis model are also available for this toxin blocking lymphocytes T action (Breland and Currier, 1983; Beeton et al., 2001).

**Table 6:** Animal toxins to treat multiple sclerosis.

Toxin/Substance	Species of origin	Effects	Experimental model	Reference
Shk	*Stichodactyla helianthus* (sea anemone)	Blocks pathological T-lymphocyte cells activation	Autoimmune encephalomyelitis rat	Norton et al., 2004
Kaliotoxin	Scorpion venom	Blocks pathological T-lymphocyte cells activation	Autoimmune encephalomyelitis rat	Breland and Currier, 1983; Beeton et al., 2001
Bee Venom	*Apis mellifera* (Honey bee)	Ameliorates disease symptoms	Autoimmune encephalomyelitis rat	Karimi et al., 2012
		Reduces inflammatory markers		
Bee Venom	*Apis mellifera* (Honey bee)	Reduces fatigue	Clinical Trials	Hauser et al., 2004; Castro et al., 2005; Wesselius et al., 2005
		Improves motor function (coordination, strength, balance)		


In some MS lesions, penetration of fibrinogen into the brain through damaged parts of the BBB contributes to the pathological process of the disease. Increased fibrin deposition on lesion sites has been reported in subjects with MS (Adams et al., 2004). Experimental evidences suggest that preventing fibrin deposition enhances nervous system regeneration capability (Akassoglou et al., 2000). Batroxobin, a toxin from the South American viper *Bothrops atrox moojeni*, reduces circulating levels of fibrinogen through conversion to an insoluble form. Treatment with batroxobin suppressed clinical signs of auto immune encephalomyelitis in rats by preventing fibrin deposition (Inoue et al., 1996; Iwai et al., 1999). Several other snake venoms are also rich in anti-fibrinogen components such as ancrod and crotalase, from the venoms of *Calloselasma rhodostoma* and *Crotalus adamanteus*, respectively, providing further research substrates for MS applications (Bell, 1997; Dempfle et al., 2000). Finally, regarding snake derived compounds, CAM-NTX, a modified derivative of cobratoxin, induces resistance to experimental allergic encephalomyelitis and reduces lymphocyte brain infiltration on a guinea pig model (Hinman et al., 1999). Altogether, this evidence suggests a possible role for snake venom therapy in MS.

BV and its components are also under investigation as drug candidates against MS. On the preclinical experimental encephalomyelitis rat model, administration of BV decreased disease symptoms and serum levels of TNF-α and nitrate (Karimi et al., 2012). Clinical evaluations of BV therapy have also been carried out with inconclusive results so far. In 2001, a clinical trial with 51 MS subjects receiving at least weekly shots of BV was completed. Although the study showed some limitations as difficulty to determine whether observed improvements came from BV therapy or periods of disease relapse, and the fact that two different sources of BV had to be used throughout the experiment, the study was completed with positive outcomes (Hauser et al., 2004). An overall analysis of symptoms improvement showed that 68% of the subjects declared some kind of positive effect from BV therapy. Most noticeable improvements were reduced fatigue, increased energy levels for everyday activities, improved balance and coordination, muscular strength, and bladder control (Hauser et al., 2004). Later on, in 2005, two other clinical studies investigated BV therapy (Castro et al., 2005; Wesselius et al., 2005). The first study had a total of nine MS subjects enrolled. Although four of the nine cases experienced some worsening of neurological symptoms, the five remaining cases informed some subjective amelioration of disease symptoms. No intense side effects were reported from BV therapy (Castro et al., 2005). The second study was a larger randomized crossover study conducted with 26 MS subjects receiving bee stings three times a week for 24 weeks (Wesselius et al., 2005). Again, BV therapy was well tolerated with no significant adverse side effects. However, the number of new gadolinium-enhancing lesions, an important diagnostic criterion for evaluating MS progression, was unchanged by BV therapy. Furthermore, relapse rate, fatigue, muscular disability and overall life quality were also unchanged after BV treatment (Wesselius et al., 2005). In sum, these data indicate that BV therapy efficacy might be dependent on the protocol used (e.g., number of shots per week), the study subjects (i.e., relapse-remitting vs. secondary-progressive cases), and the models used. Although side effects have not been a concern, it cannot be ruled out as limitations for BV therapy until further data from larger studies are collected.

## Perspectives for Future Use of Animal Toxins

Venoms are fruitful natural sources of new molecules, which have been relentlessly enhanced by evolution through natural selection. Generally, venom components possess peculiar characteristics such as low-molecular mass, stability, high potency, apart from selectivity and affinity for a wide variety of targets in mammalian systems (Jones and Bulaj, 2000; Casewell et al., 2013). The studies discussed in this review highlight toxins exhibiting potential for either decreasing or even inhibiting the progression of neurodegenerative processes. However, despite all these studies about the application of animal toxins as therapeutic tools to treat neurodegenerative processes, few molecules were tested in clinical trials. In this review, we mentioned BV clinical trials in subjects with PD (Cho et al., 2012; Hartmann et al., 2016) and SNX-111 in subjects with cerebral ischemia (Heading, 2001; Smith and Deer, 2009). The low representativeness of these studies is due to several reasons. First, the scientific evidences showing benefits of a particular toxin in a neurodegenerative disease are not, in most cases, sufficiently strong to justify the studies in humans. Most of the studies described are still preliminary, and often were performed without the correct controls and/or using just one animal model. Among the studies that have compared the effectiveness of a toxin and another drug used as positive control, we could highlight the studies investigating BV and the dopamine agonist pramipexole in a PD model (Kim et al., 2011); a study that compares BVPLA_2_ with donepezil, a FDA-approved drug that inhibit cholinesterase in an AD model (Ye et al., 2016b); as well as studies comparing the performance of *Phoneutria nigriventer* toxins to *Conus* toxins in ischemia models (Pinheiro et al., 2006, 2009). Second, the animal models employed to study neurodegenerative diseases possess several limitations, mainly due to our restricted knowledge about disease etiology (Dauer and Przedborski, 2003; Jucker, 2010). Most neurodegenerative diseases are a consequence of sporadic factors or the sum of neuropathological events, making it difficult to develop an animal model that recapitulates all the complex clinical features of human diseases. For instance, most animal models are generated through genetic mutation, recapitulating familial neurodegenerative diseases, which only represent a small fraction of the cases. Thus, without employing reliable animal models, it is difficult to select and test toxins to be used in clinical trials. The third reason for this lack of success is that many of the studies described here used crude venom without evaluating the compounds in isolation, which restricts the knowledge about the neuroprotective mechanisms and hinder studies aiming to enhance compound pharmacological properties. In most cases, the methodological approach to separate the different compounds present in venom can be daunting. In addition, we have to consider the difficulty to obtain biologically active fractions. Research involving animal venoms is not a trivial task and there are many bioanalytical challenges to overcome, as well as the intrinsically time-consuming process of elucidation the occurrence of synergistic actions between components present in the poison (Bliss, 1939). The fourth reason is that the amount of venom obtained from animals is insufficient to perform all the necessary experiments and tests. As mentioned previously, venoms are extremely potent and, thus, small amounts are sufficient for animals’ need, such as defending themselves from predators or attacking prey. Although most research with animal toxins uses native proteins that are obtained directly from animals, an alternative approach would be synthesis either by recombinant expression or chemical production (Zhao et al., 1994; Perez-Pinzon et al., 1997; Lu et al., 2016; Smith et al., 2016).

Recombinant proteins are produced by employing the recombinant DNA technique, whereby the DNA sequence encoding the protein of interest is inserted into a plasmid and subsequently introduced into a host organism, such as bacteria or yeast, which will be induced to express the gene of interest. This method enables the production of proteins that are the same or similar to the original, facilitating the acquisition of large quantities and/or with superior activity (Cohen et al., 1973; Harris et al., 1986; Kopetzki et al., 1989). However, production of recombinant proteins is not an easy assignment and depends on the proper selection of suitable hosts and vectors. Besides, there is no guarantee to obtain a structurally active protein at the end of the process. Toxins are molecules that possess many cysteine and disulfide bridges that are important for protein proper folding, which makes their production even more difficult with aggregation often limiting the yield of properly folded proteins (Fahnert, 2012). These problems occur because, in most cases, the primary host of choice for the production of recombinant proteins is bacteria, mainly *Escherichia coli*. Undoubtedly, the production of recombinant proteins in prokaryote systems has revolutionized biochemistry. Bacterial protein expression systems are popular because they are easy to culture, fast growing and yield large quantities of recombinant protein. However, if eukaryotic post-translational modifications (like disulfide bridges) are indispensable for protein folding and activity, a prokaryotic expression system may not be convenient (Rosano and Ceccarelli, 2014; Stefan et al., 2015). In that case, mammalian or insect cells may be a more suitable system for recombinant protein production. These systems are able to facilitate protein proper folding and enable post-translational modifications, which are important for full biological activity. Despite these advantages, these expression systems are not widely used due to their high cost, complicated technology, and potential for contamination with proteins from mammalian cell viruses (Khan, 2013). Chemical synthesis of proteins, especially using established solid-phase techniques is a fast and effective technique, and can be employed to overcome some of the disadvantages of current protein production methods using cell systems (Marglin and Merrifield, 1970; Borgia and Fields, 2000; Kent, 2003). However, this methodology also has its limitations, such as aggregation of growing peptide chains, numerous secondary reactions and low yields of long peptides (>25–30 residues) (Nilsson et al., 2005).

Despite all the limitations of the studies performed and the difficulties in obtaining toxins, either from the native source or by artificial methods, their neuroprotective abilities are undeniable. Moreover, many drugs approved during the past decades are based on animal’s toxins or their compounds. Some successful examples are Prialt^®^ (ziconotide), which is a synthetic version of ω-conotoxin MVIIA found in the venom of the fish-eating marine snail, *Conus magus*, and used to treat severe chronic pain (Heading, 2001; McGivern, 2007); angiotensin I-converting enzyme inhibitors, derived from the venom of the South American Lancehead snake (*Bothrops jararaca*), which was the lead compound used for the development of anti-hypertensive drugs such as Captopril^®^ and its analogs (Rocha et al., 1949; Ferreira et al., 1970; Cushman and Ondetti, 1980); the inhibitor of platelet aggregation drug Aggrastat^®^ (tirobifan), derived from the venom of the saw scaled viper *Echis carinatus* (Garsky et al., 1989); Integrilin^®^ (Eptifibatide), an anticoagulant drug derived from the venom of the southeastern pygmy rattlesnake (*Sistrurus miliarius barbouri)* (Platelet Glycoprotein IIb/IIIa in Unstable Angina: Receptor Suppression Using Integrilin Therapy (PURSUIT) Trial Investigators, 1998; Hashemzadeh et al., 2008); and Byetta^®^ (exenatide) derived from the venom of the Gila monster (*Heloderma suspectum*) and used as treatment for diabetes type II (Nielsen et al., 2004; Triplitt and Chiquette, 2006). Therefore, it is possible that in the near future animal toxins might offer new and effective therapeutic options, facilitating the development of disease-modifying drugs to treat neurodegenerative diseases.

## Author Contributions

JdS and BG helped design the study and wrote the manuscript. FR and LV drafted the study and substantially contributed to the conception of the work. FR, LV, and MG revised this study critically for important intellectual content. All authors approved the final version of the manuscript.

## Conflict of Interest Statement

The authors declare that the research was conducted in the absence of any commercial or financial relationships that could be construed as a potential conflict of interest.

## Abbreviations

Aβ: amyloid beta
AChE: acetyl cholinesterase
AD: Alzheimer’s Disease
ALS: amyotrophic lateral sclerosis
AMPA: α-amino-3-hydroxy-5-methyl-4-isoxazolepropionic acid
APP: amyloid precursor protein
ASIC: acid-sensing ion channel
BBB: blood-brain-barrier
Bmk: Buthus martensii kirsch
BV: bee venom
BVPLA_2_: bee venom phospholipase A2
CNS: central nervous system
ECE-1: endothelin-converting-enzyme-1
FDA: Federal Drugs Administration
GFP: green fluosrescent protein
HD: Huntington’s Disease
HWTX-I: huwentoxin-I
i.c.v.: intracerebroventricular
i.p.: intraperitoneal
IFNβ: interferon-β
IL-1: interleukin-1
iNOS: inducible nitric oxide synthase
LPS: lipopolysaccharide
MAC-1: macrophage antigen complex-1
MAO-B: monoamine oxidase-B
MCA: middle cervical artery
MDA: malondialdehyde
mGluR: metabotropic glutamate receptor
MPP+: 1-methyl-4-phenylpyridinium
MPTP: 1-methyl-4-phenyl-1,2,3,6-tetrahydropyridine
MS: multiple sclerosis
NEP: neprilysin
NFT: neurofibrillary tangles
NMDA: *N*-methyl-D-aspartate
ODLG: oxygen deprivation low glucose
PcTX: psalmotoxin-1
PD: Parkinson’s Disease
PGE2: prostaglandin E2
PLA_2_: phospholipase A2
PON1: paraxonase-1
PS: presenelin
RGCs: retinal ganglion cells
ROS: reactive oxygen species
RVV-V: Russian viper venom factor V
SNpc: substancia nigra pars compacta
SOD: superoxide dismutase
SSM: *Scolopendra subspinipes mutilans*
SVHRP: scorpion venom heat resistant peptide
TNFα: tumor necrosis factor α
UPS: ubiquitin proteasome system
VSSC: voltage sensitive calcium channel
WT: wild type
